# Opening of the Blood-Brain Barrier before Cerebral Pathology in Mild Hyperhomocysteinemia

**DOI:** 10.1371/journal.pone.0063951

**Published:** 2013-05-16

**Authors:** Bryce C. Rhodehouse, Jamie N. Mayo, Richard S. Beard, Cheng-Hung Chen, Shawn E. Bearden

**Affiliations:** 1 Department of Biological Sciences, Idaho State University, Pocatello, Idaho, United States of America; 2 ISU Biomedical Research Institute, Idaho State University, Pocatello, Idaho, United States of America; INSERM, France

## Abstract

Hyperhomocysteinemia (HHcy) is a risk factor for cognitive impairment. The purpose of this study was to determine the temporal pattern of cerebral pathology in a mouse model of mild HHcy, because understanding this time course provides the basis for understanding the mechanisms involved. C57Bl/6 mice with heterozygous deletion cystathionine β-synthase (*cbs* ^+/−^; Het) were used as a model of mild HHcy along with their wild-type littermates (*cbs* ^+/+^; WT). Mice were ‘young’ (5.3±0.2 months of age) and ‘old’ (16.6±0.9 months of age). Blood-brain barrier (BBB) permeability was quantified from Evans blue and sodium fluorescein extravasation. Microvascular architecture was assessed by z-stack confocal microscopy. Leukoaraiosis was measured from Luxol fast blue stained slides of paraffin brain sections. Inflammation was quantified using standard antibody-based immunohistochemical techniques. Cognitive function was assessed using the Morris water maze. BBB permeability was significantly greater in Het vs. WT mice at all ages (p<0.05). There were no differences in microvascular architecture among the groups. Compared with all other groups, old Het mice had significantly greater leukoaraiosis, inflammation in the fornix, and cognitive impairment (p<0.05). In mild HHcy, increased permeability of the BBB precedes the onset of cerebral pathology. This new paradigm may play a role in the progression of disease in HHcy.

## Introduction

Hyperhomocysteinemia (HHcy) is an elevation of plasma levels of the aminothiol homocysteine (Hcy), which is formed during methionine metabolism. HHcy is a risk factor for cerebrovascular disease and cognitive impairment [1], [2]. Mild to moderate HHcy occurs in 5–10% of the general population, 40% of patients with peripheral vascular disease, and may reach greater than 90% in patients on hemodialysis [3]–[5]. HHcy can be caused by dietary loading (e.g., high methionine diet) and insufficiency in the activity of enzymes involved in regulating Hcy levels, such as lack of vitamin cofactors, side effects of pharmaceuticals (e.g., metformin and L-dopa), or genetic mutations of the enzymes. Remethylation depends on vitamin B_9_ and B_12_ while transsulfuration requires vitamin B_6_. Transsulfuration is the only pathway for permanent disposal of Hcy because this pathway is not reversible in humans (remethylation cycles between Hcy and methionine). In the elderly, dietary deficiencies may reduce intake of B_6_, B_9_, and B_12_. This dietary narrowing may be the primary reason for the increase in homocysteine with aging. Genetic deficiencies of the enzymes in these two pathways may be common (e.g., point mutations in cystathionine β-synthase). In rare forms of enzyme deficiencies, severe neurological conditions (e.g., stroke) can occur even in infants but the extent to which the more common deficiencies cause clinically relevant elevations in Hcy is not established [6].

Redox imbalance is thought to cause much of the cerebrovascular dysfunction associated with HHcy [7]. Disruption of the blood-brain barrier (BBB) in rodents [6], [8]–[10] and humans [11] is among the most striking homeostatic disturbances resulting from HHcy (vascular complications of HHcy are reviewed in [6], [12]). In rodent models, even a subclinical elevation in Hcy levels (e.g., from baseline of ∼3 µmoles/L to ∼12 µmoles/L) results in significant leak of plasma proteins into the brain that include albumin [9], [10] and endogenous immunoglobulins [6]. These results are consistent with the finding that HHcy contributes to subcortical damage, infarcts [13]–[15], and progressive cognitive impairment [1], [16], [17].

Epidemiological studies demonstrate that the onset of HHcy precedes the development of cognitive impairment (reviewed in [2]). Collectively, these studies implicate HHcy in the etiology of neuropathology but there remains a critical gap in knowledge in the field. Specifically, it remains unknown how HHcy temporally and morphologically manifests in BBB opening, tissue damage, and cognitive impairment. The purpose of this study was to establish the time-course of these cerebral pathologies thought to contribute to cognitive impairment using a mouse model of mild HHcy.

## Materials and Methods

### Ethics Statement

All procedures were approved by the Institutional Animal Care and Use Committee of Idaho State University and performed in accord with the National Institutes of Health Guide for the Care and Use of Laboratory Animals.

### Murine Model

*Cbs^+/−^* mice (Het) and wild-type (WT) littermates were studied as adults (5.3±0.2 months of age; n = 79; 26.3±0.5 g) and in old age (16.6±0.9 months of age; n = 34; 33±1.1 g). Founders for our colony were provided to us by Dr. Steven Lentz, University of Iowa in 2008, and have been backcrossed at least 15 generations on the C57Bl/6 background. Animals are housed on a 12/12 hour light/dark cycle with food and water ad libitum. We have previously published a comprehensive review of this well established mouse model of mild HHcy [6] and an excellent review of different murine models of HHcy has been published by the Lentz group [12].

### Quantification of Plasma Hcy Concentrations

Total plasma Hcy concentrations were measured in samples collected from each mouse by cardiac puncture at the time of euthanasia using a commercial enzyme immunoassay kit, Homocysteine-EIA kit (BioRad), as previously described [8] and according to the manufacturer’s directions.

### Brain Collection and Histology For Leukoaraiosis

Brains were perfusion fixed through a catheter in the left ventricle (effluent via the right atrium) with 4% fresh paraformaldehyde in PBS, harvested, and embedded in paraffin. Paraffin embedded brains were sectioned (5–7 µm) in the coronal plane and stained with Luxol fast blue to visualize myelin. Brightfield images were analyzed using ImageJ software (National Institutes of Health). The extent of leukoaraiosis was quantified as previously described [18] by investigators blinded to the ages/genotypes: 0 = no lesions, 1 = focal lesions, 2 = beginning confluence of lesions, 3 = diffuse, confluent lesions involving the entire region.

### BBB Permeability

Permeability of the BBB was quantified by Evans blue and sodium fluorescein extravasation using standard techniques. *Cbs^+/−^* mice and WT littermates were injected with Evans blue dye (i.p.; 3% in 100 µl sterile PBS). Two hours later, mice were anesthetized with isoflurane; blood was collected from the left ventricle into plasma separator tubes followed by transcardial perfusion to flush remaining blood from cerebrovasculature. Brains were rapidly harvested, dissected into right and left hemispheres, and weighed. One hemisphere was homogenized on ice with a dounce homogenizer (6 strokes) in 0.5 ml ice-cold PBS followed by centrifugation at 12,000 g for 15 min at 4°C. Supernatants were used for fluorescence measurements. Evans blue extravasation was calculated by subtracting background fluorescence and then dividing the brain fluorescence (excitation = 540 nm, emission = 680 nm) by plasma fluorescence and normalizing to brain weight. In the other hemisphere, epifluorescence of brain sections was imaged with a fluorescence imager (BioRad). BBB permeability was also assessed with sodium fluorescein (NaF; ∼400 Da) as for Evans blue with the following differences. Intraperitoneal injections were with 10 mg NaF in 0.1 mL sterile saline. Protein was precipitated from brain and serum samples with trichloroacetic acid (TCA) to remove potential background fluorescence. To prevent precipitation of NaF in serum, the samples were diluted 1∶10 in sterile PBS prior to an additional 1∶10 dilution in 20% TCA. Supernatants from brain homogenates were diluted 1∶10 in 20% TCA. All samples were incubated at 4°C for 24 h. Samples were centrifuged at 10,000 g for 15 min to remove precipitated protein. The supernatant was removed and diluted with equal volumes of borate buffer (0.05 M, pH 10), resulting in a final concentration of 10% TCA and 0.025 M borate buffer to neutralize pH. Fluorescence of NaF was quantified as an indicator of BBB permeability using a fluorescence plate reader, (excitation 480/20 nm, emission 538/20 nm; Synergy HT, BioTek) and expressed as relative units (RU) of fluorescence per gram of brain tissue normalized to plasma fluorescence (Brain:serum fluorescence = RU brain fluorescence/RU plasma fluorescence/brain weight (g)).

### Cognitive Function: Spatial Memory and Navigation

Spatial memory was assessed and quantified using the Morris water maze [19]. A circular pool 1.82 m in diameter and 0.9 m deep was filled to 12 cm from the top with 24°C water made opaque with off-white tempera paint. A circular escape platform 20 cm in diameter was submerged 0.5 cm below the surface of the water. Three 22×28 cm posters with different symbols were placed on the room walls (cinderblock walls painted white) to serve as visual cues. The mice underwent two days of training with the platform visible just above the water surface before training began. Mice were placed in the pool at the wall and given 60 sec to find the platform. If the mouse failed to reach the platform in 60 sec, they were coaxed to it. After reaching the platform, mice were given 10–15 sec to orient before being rescued, dried, and returned to their cages. Learning Trials: To assess the acquisition of spatial memory, mice were trained for 4 days with 4 training swims each day and approximately 30 min between swims. The platform remained in the same position for all of the learning trials. Probe Trials: To further assess spatial memory and navigation, a probe trial was performed on days 2–4 in which the platform was removed from the pool; mice were allowed to search for the platform for 60 sec. All trials were video recorded for analysis. All swims were recorded with a digital camera suspended over the pool and videos were analyzed using a computer tracking program written by us (co-author CHC).

### Immunohistochemistry

Immunohistochemistry was performed using standard methods. Brain sections were blocked overnight at 4°C with respective antibodies. Antibodies used: anti-leukocyte common antigen (CD45; BioLegend; 7 µmoles/L), anti-vascular cell adhesion molecule-1 (VCAM1; Santa Cruz Biotech; 7 µmoles/L). The anti-beclin-1 caspase cleavage product (BeclinCCP) was a gift of Dr. Troy Rohn (Boise State University) [20]. VCAM1 is expressed by activated endothelium and plays an important role in recruiting leukocytes during the inflammatory process. CD45 is a general marker of leukocytes that indicates an ongoing inflammatory response by the immune system. Sections were washed for two hours in TBST then incubated with secondary antibody at a dilution of 1∶500 in TBST. For microvascular architecture, vessels were labeled using thick (∼1 mm) free floating fresh slices stained overnight at room temperature with isolectin conjugated with AlexaFluor 488 (Invitrogen). Microvascular architecture, including arterioles, venules, and capillaries, was quantified for a voxel of 630×630 µm^2^ through an 85 µm^2^ z-stack. Images were acquired by fluorescence microscopy (Leica, DMLFS) with digital camera (MicroPublisher 3.3 RTV; QImaging; QCapture 5.1 software) using a 20X/NA 0.4 or 40X/NA 0.55 objective or an Olympus FV1000 laser scanning confocal microscope at 512×512 resolution using a 10X/NA 0.4 or 40X/NA 0.9 objective. Images were analyzed with Image J or Metamorph software.

### Statistics

GraphPad Prism 6 was used for analyses. Pair-wise comparisons were performed with student t-test and group-wise data were compared using analysis of variance with Tukey or Dunnett post-hoc analyses. Alpha was set at 0.05 for statistical significance prior to the start of experiments. All data are presented as the mean ± standard error of the mean.

## Results

**Figure 1:**
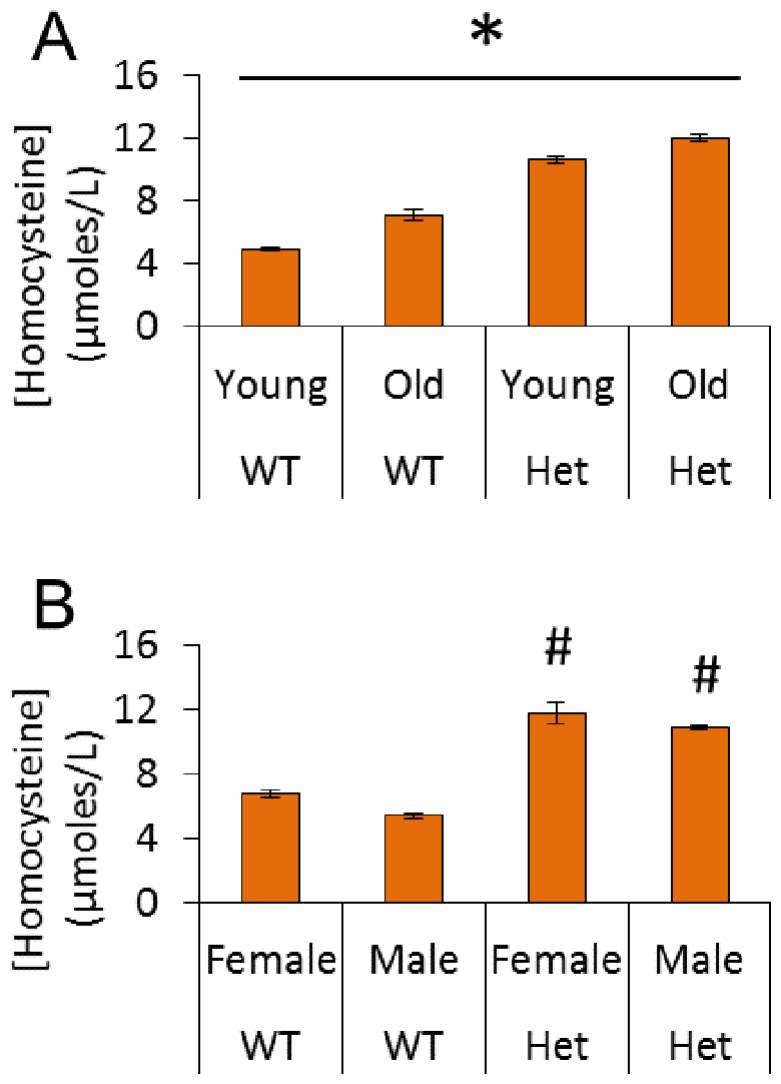
Plasma levels of homocysteine. A) Homocysteine levels by age and genotype; *p<0.05 for all pairwise comparisons indicating that there was a significant increase in homocysteine levels with age for both groups and that homocysteine levels were higher for cbs^+/−^ mice than WT mice.B) Homocysteine levels by sex and genotype; ^#^p<0.05 vs. WT groups.

Hcy levels were significantly higher in Het vs. WT mice and increased with age in both groups (Fig. 1A). While Het mice had significantly higher Hcy levels than WT, there was no difference between male and female mice within each genotype (Fig. 1B). These values are consistent with previous studies of this model [6], [12] and match the elevation of Hcy levels in the general population (10–15 µM). However, these values are boarder line for clinical of HHcy which is generally diagnosed for patients with levels >12–15 µM followed by moderate HHcy (30–100 µM) and severe HHcy (>100 µM). Hence, this study is aimed at understanding the subclinical and borderline clinical elevations in Hcy common in the general population.

**Figure 2:**
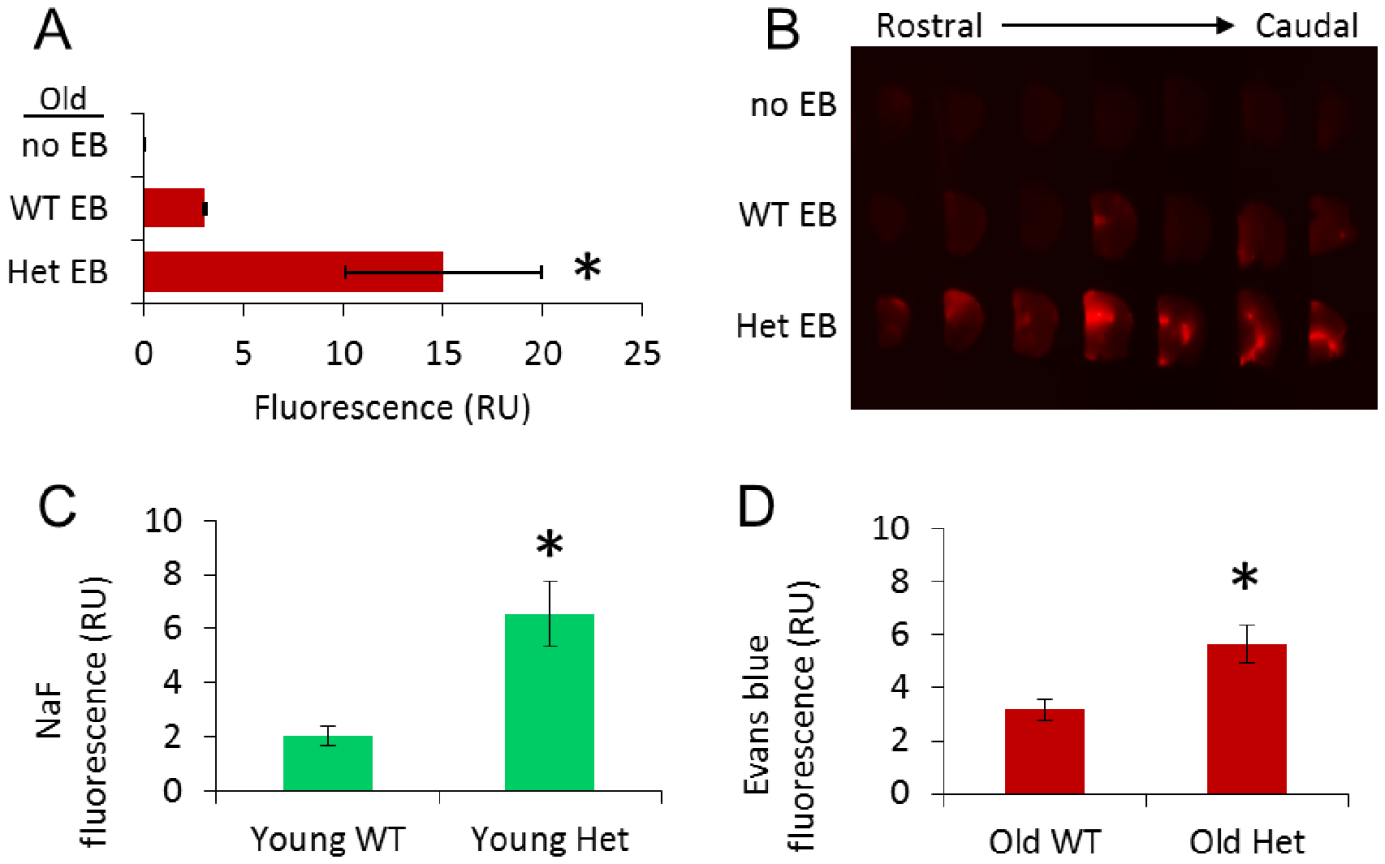
Permeability of the BBB. A) Quantification of BBB extravasation of Evans blue (EB) in old mice. *p<0.05 vs. other groups B) Representative image showing serial sections (1 mm) from rostral to caudal from the brain of a mouse without Evans blue injection (no EB), in wild type mouse (WT; *cbs^+/+^*) with Evans blue injection, and heterozygous hyperhomocysteinemic mouse (Het; *cbs^+/−^*) with Evans blue injection. BBB extravasation of C) sodium fluorescein in young mice and D) Evans blue in old mice. *p<0.05 vs. WT.

**Figure 3:**
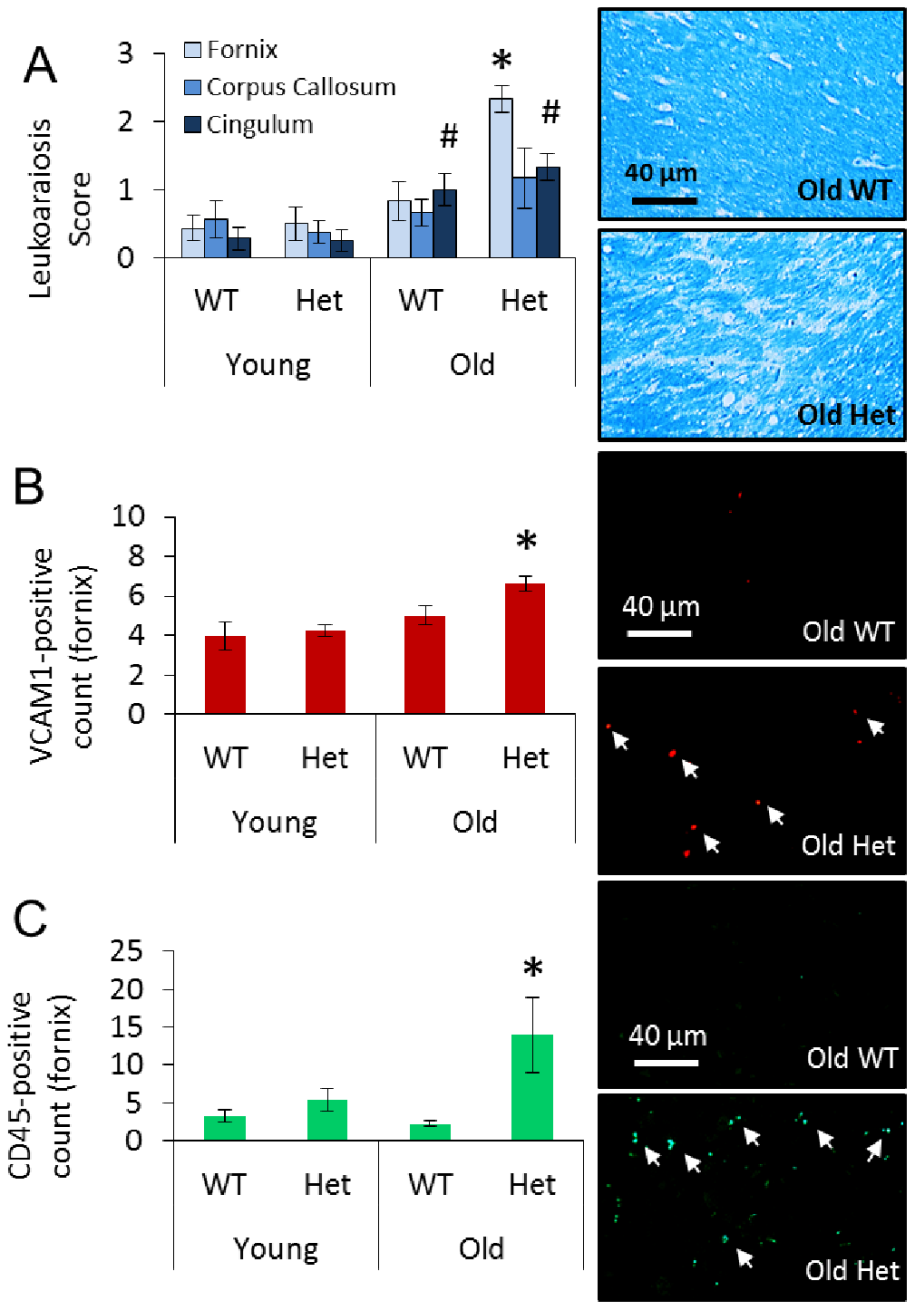
White matter histology. A) Leukoaraiosis in fornix, corpus callosum, and cingulum with representative images of Luxol fast blue staining in fornix. *p<0.05 vs. all other groups within region, ^#^p<0.05 vs. young groups within region. Inflammation in fornix shown by B) VCAM1 and C) CD45 positive labeling with representative images of each and examples of positive labeling indicated by arrows. *p<0.05 vs. all other groups.

**Figure 4:**
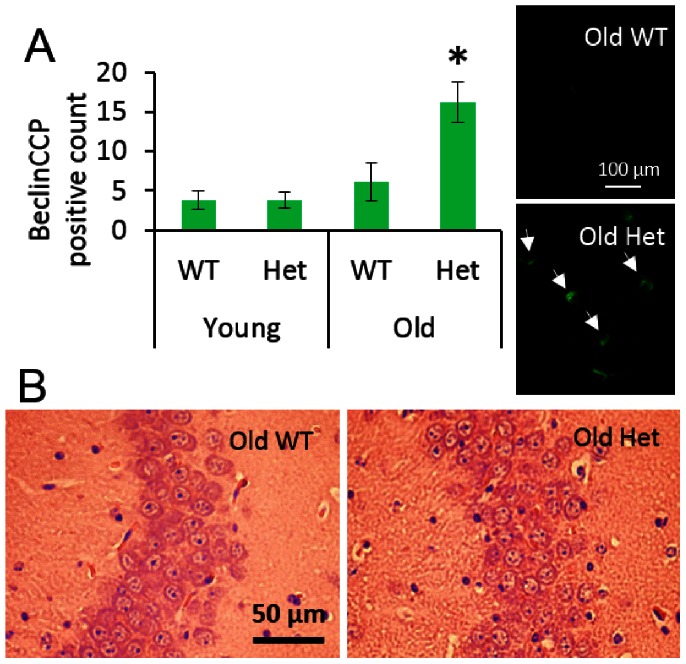
Hippocampal histology. A) Count of cells that are positive for the caspase cleavage product of beclin-1 (BeclinCCP) with arrows demonstrating positive cells. *p<0.05 vs. all other groups B) Representative images of H&E stained pyramidal layer and surrounding extrapyramidal tissue. H&E analyses did not reveal gross pathology, suggesting cognitive impairment occurs from neuronal dysfunction more than neuronal loss.

Permeability of the BBB was greater in the Het vs. WT groups at all ages (Fig. 2). The increased permeability was found particularly throughout the subcortical regions, hippocampus, and white matter (Fig. 2B). In the old Het group, the same regions with high permeability showed significant histopathology. Leukoaraiosis was significantly greater in the fornix for the old Het vs. old WT groups (Fig. 3A). There was no significant leukoaraiosis in the young mice for either WT or Het. In the cingulum, the extent of leukoaraiosis score was significantly greater in old vs. young (age effect). The amount of inflammation, as indicated by VCAM1 and CD45 immunoreactivity, was greater in the old Het vs. all other groups (Fig. 3B–C). As indicated by positive labeling for the caspase cleavage product of beclin-1 (BeclinCCP), the number of cells with disrupted autophagy was significantly greater in the old Het vs. all other groups (Fig. 4A). The BeclinCCP positive cells were consistently distributed throughout the hippocampus and in the dentate gyrus with no apparent pattern of regional bias except that the majority, but not all, of immunopositive cells were in extrapyramidal regions. H&E staining did not reveal appreciable differences in neuronal morphology that would suggest cell death (Fig. 4B). There were no significant differences between Het and WT groups for any of the microvascular architectural measurements (Table 1): total vessel length, average vessel length, average diameter, total surface area, number of segments, total vessel volume, average vessel volume, and vascular density.

**Table 1:** Microvascular morphology.

	Young		Old	
	WT	Het	WT	Het
Total Length (µm)	24,367±589	24,357±1,054	20,772±2,979	27,834±2,527
Average Segment Length (µm)	21±2	19±1	22±3	17±1
Average Diameter (µm)	7±0.3	7±0.3	8±0.4	7±0.3
Total surface area (µm^2^)	498,309±20,636	517,109±41,961	493,986±81,175	627,173±79,941
Segments (count)	1,172±80	1,301±90	1,089±256	1,696±203
Total Volume (µm^3^)	158,617±6,569	164,601±13,357	157,241±25,839	199,636±25,446
Average Segment Volume (µm^3^)	141±17	126±2	165±18	117±2
Vascular Density (%)	10±0.4	10±1	10±2	12±2

No significant differences.

**Figure 5:**
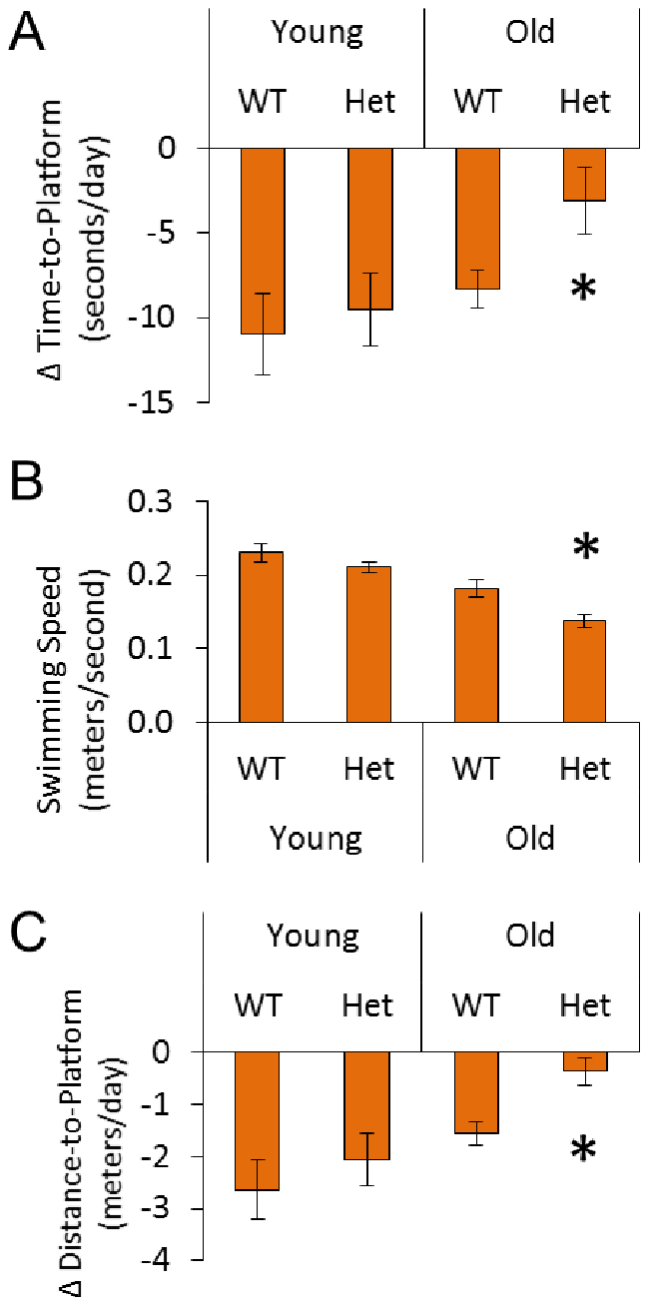
Learning Trials for Morris water maze. Mice learned to find a platform submerged below water level over the course of four days and four trials per day. A) The average daily improvement for each mouse in the time taken to reach the platform (often termed escape latency). A negative value means a shorter time; for example, young WT mice reduced their time-to-platform by more than 10 seconds per day. B) Average swimming speed. Because there were significant differences in swimming speed, data were then reevaluated following normalization. C) Performance normalized for differences in swimming speed and represented by the average daily improvement in the distance travelled to reach the platform for each mouse. For all panels *p<0.05 vs. all other groups.

**Figure 6:**
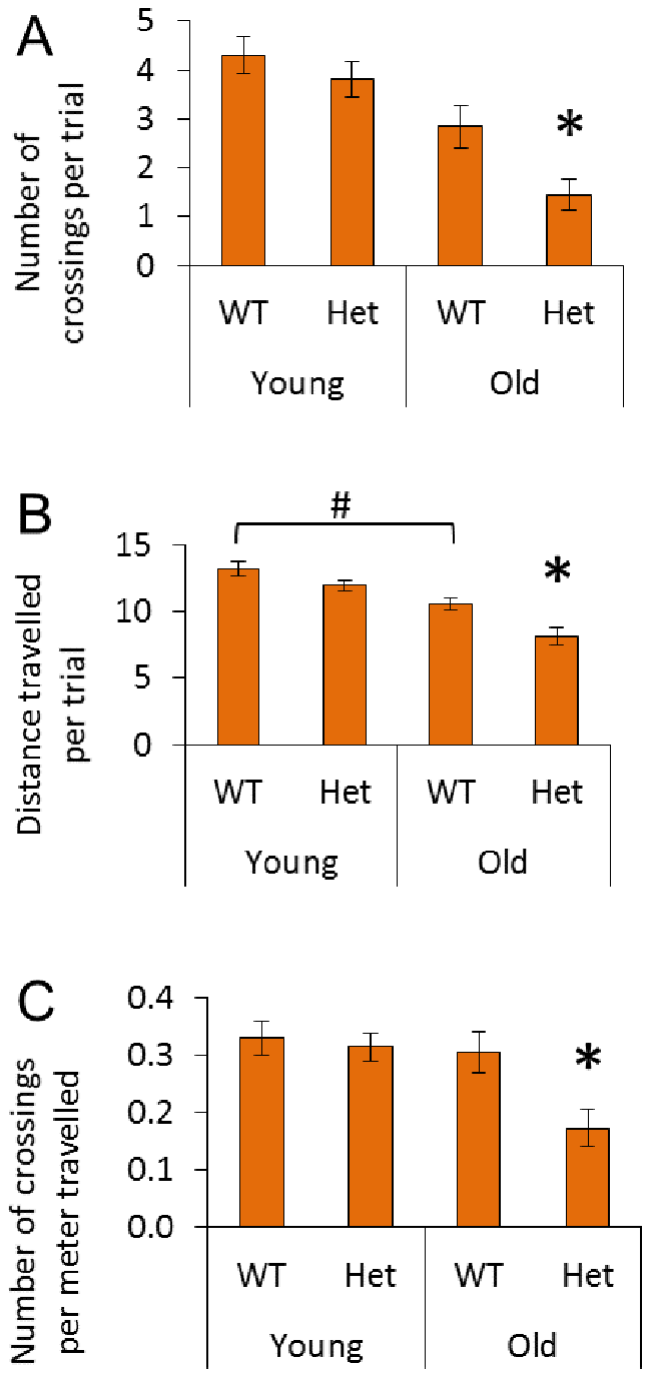
Probe Trials for Morris water maze. Following learning trials on days two through four, the platform was removed. Mice were allowed to attempt finding the platform location for 60 sec. A) Number of times each mouse correctly crossed the area where the platform had been. *p<0.05 vs. all other groups for all three panels. B) Total distance travelled during the 60 sec in search of the escape platform. ^#^p<0.05 young WT vs. old WT. Because there was a significant difference in the distance travelled per trial, owing to differences in swim speed (figure 5), data were then reevaluated following normalization. C) Number of times in 60 sec that mice correctly swam over the previous platform site normalized to the distance travelled. *p<0.05 vs. all other groups.

We used the Morris water maze to test spatial learning and navigation. During initial familiarization days mice located an elevated, visible platform. On these days there were no differences among groups in the ability to locate the visible platform (data not shown). Therefore, differences in performance on subsequent learning and probe trials are unlikely to be due to differences in visual acuity. During the four days of learning trials, when the mice performed four swims per day to find the submerged escape platform, mice were expected to perform the task faster each day as they learn the platform location. Old Het mice improved their performance time significantly less than the other groups across the four day indicating an impaired capacity for spatial learning and cognition (Fig. 5A). The swimming speed of the old Het mice was less than that of any other group (Fig. 5B). This may result from motor dysfunction and/or impaired cognitive processing. To confirm that there is slow cognitive processing in old Het mice, we normalized the data for each mouse to their respective swimming speeds; old Het mice persisted with significantly poorer performance than any other group confirming impaired spatial learning and cognition (Fig. 5C). At the end of days two through four, the platform was removed and mice were challenged to find the missing platform for 60 sec. Again old Het mice performed significantly more poorly than the other groups, failing to cross the platform site as many times as mice in the other groups (Fig. 6A). Due to the slower swimming speeds, both aging and agingXgenotype (old Het) swam a shorter total distance than younger counterparts (Fig. 6B). Therefore, we normalized the number of platform crossing to the distance travelled to check whether impaired performance might be attributable to the shorter distance travelled. This was not the case as old Het mice failed to correctly locate the platform site as many times as the other three groups even as a function of the distance travelled (Fig. 6C) which confirms significant impairment of spatial memory and cognitive performance with aging in HHcy.

## Discussion

In HHcy, cognitive dysfunction ranges from mild cognitive impairment to frank dementia [1], [2], [21]. This study elucidates the progression of cerebral pathology in mild HHcy demonstrating that young *cbs^+/−^* mice are free from the cerebral and cognitive pathologies measured with the exception of BBB permeability, while old *cbs^+/−^* mice have cognitive impairment along with a multitude of cerebral histopathologies. These results suggest there may be a therapeutic or physiologic window where targeted treatments or life-style factors may influence the outcomes of HHcy. However, the results do not rule out the possibility that HHcy simply sets the process in motion and lowering Hcy levels after they have been elevated may not reduce disease development. Specifically, these results show an increase in the permeability of the BBB months before the onset of neuroinflammation, leukoaraiosis, and cognitive dysfunction. In many clinical trials, lowering Hcy levels with B-vitamin supplementation has not been effective in reversing the pathologies associated with HHcy. In this study, young HHcy mice had significant BBB permeability but did not show signs of histopathology or cognitive impairment. It was not until later in life that neurological pathologies developed. It is possible that secondary prevention has met with mixed or poor results because the BBB pathology has progressed too far for consistent effects of lowering Hcy.

The BBB is a selectively permeable and regulated interface between the blood and the neurons and glia of the brain. The BBB is formed by endothelial cells in concert and cooperation with other cells of the neurovascular unit, especially pericytes and astrocytes. Increased permeability of the BBB appears to play a role in the pathogenesis of several neurological diseases including vascular cognitive impairment and stroke [22]. Mounting evidence implicates an increase in the permeability of the BBB in the etiology of cerebral small vessel disease, white matter lesions or leukoaraiosis, and non-embolic stroke [23], [24]. In this context, the prevailing theory is that loss of BBB integrity leads to dysfunction due to disruption of the neurovascular unit. For example, an increase in BBB permeability was observed prior to senile plaque formation in an Alzheimer’s disease model [25] and has been implicated in the etiology of cognitive decline in type 2 diabetes [26]. In the spontaneous hypertensive rat model, intracerebral hemorrhage was preceded by an increase in the permeability of the BBB [27]. In humans, HHcy and increased BBB permeability are each independent risk factors for lacunar infarcts and leukoaraiosis [23], [28], [29]. There may also be a connection among BBB breakdown, myelin loss, and neuroinflammation in hypertension [30].

An important result in our studies is that young mice with chronic, genetic HHcy did not show cognitive impairment based on the assessments made. Thus, the development of histopathology and cognitive decline with age are suggested to be long-term consequences of BBB leak over a significant portion of the lifespan rather than an acute outcome, which has been inferred from studies using different models. For example, a previous study used a B-vitamin deficient diet as a means to cause HHcy in experimental mice [31]. Similar to our current (Fig. 5–Fig. 6) and recent results [32], the animals developed cognitive impairment. However, the authors also reported a concomitant and significant inverse correlation between hippocampal capillary length and escape latency on Morris water maze. They speculated that the cognitive impairment in B-vitamin deficiency HHcy may be caused by microvascular rarefaction. Due to the ubiquitous importance of B-vitamin for myriad cellular functions, there are limitations in the interpretation of this model because the effects of B-vitamin deficiency may mask the effects of Hcy or its metabolic pathways, per se [12]. Many of these are overcome in the *cbs*-deficient model [6]. Our results, in the heterozygous *cbs*-deficiency model of HHcy did not provide evidence of significant microvascular rarefaction or of alterations in any of the measures of microvascular architecture (Table 1). It is important to keep in mind that flux across the BBB is influenced both by the flux at any given point and the total surface area for that flux in a given brain region. Results from the architectural analyses of hippocampal microvasculature demonstrate that a change in surface area for diffusion is not the mechanism for increased BBB permeability in HHcy. This is consistent with our previous studies demonstrating uncoupling of adherens and tight junctions of cerebral endothelium in HHcy [6], [33]. However, our conclusions are similar to those of Troen et al. [31] because our results are consistent with the idea that HHcy first causes a microvascular pathology, a cerebral small vessel disease with BBB opening, that then progresses to leukoaraiosis and cognitive dysfunction [34].

Beclin-1 is the mammalian orthologue of yeast Atg6 and is a critical factor in the initiation of autophagy. Autophagy is required for cellular homeostasis, especially in response to stress. Beclin-1 plays a fundamental role in modulating the balance between autophagy and apoptosis. Cleavage and inactivation of beclin-1 by caspase-3, producing the cleavage product detected by the antibody we used, is a marker for dysfunctional regulation of homeostasis by reduced autophagy and is associated with neurodegeneration [35], consistent with the complementary results of leukoaraiosis and inflammation in our studies. This pathway promotes apoptosis several ways: inhibition of autophagy reduces the degradation of pro-apoptotic caspase 8, degradation of beclin-1 impairs its ability to block activation of pro-apoptotic Bid, and cleaved fragments of beclin-1 translocate to mitochondria and stimulate apoptosis [35], [36]. Others have suggested that HHcy initiates mitophagy [37]. Here we uncover the possible mechanistic explanation that HHcy stimulates the transition from homeostatic autophagy to neuronal dysfunction by disrupting the beneficial control of the cellular autophagy stress response through caspase cleavage of beclin-1 (Fig. 4). Inspection of H&E stained hippocampi did not reveal any appreciable gross degenerative changes, suggesting the dysfunction is manifest through impaired neuronal function and possibly specific cellular degeneration rather than widespread neuronal apoptosis. This is consistent with the results of the Morris water maze tests where the old HHcy mice showed significant impairment but not a profound loss of spatial learning and memory capacity; they were slower and less accurate but were generally still able to learn the platform location.

In the white matter tracks of the fornix, leukoaraiosis was clearly evident in the older HHcy group by loss of Luxol fast blue staining (Fig. 3A). In humans, the association between HHcy and leukoaraiosis is well documented [1], [13], [14], [29], [38], [39]. Our results showing an increase in markers of inflammation, VCAM1 and CD45 (Fig. 3B–C), follows a similar paradigm to that documented in other progressive neurological diseases that correlate BBB leak with cognitive impairment [22]. This inflammation occurs without evidence of changes in the levels of BBB transporters of amyloid-beta [32] and, therefore, appears to be a separate pathology from the current amyloid-beta paradigm of Alzheimer’s disease progression (i.e., a vascular cognitive impairment).

The fornix is an accumulation of afferent and efferent myelinated axons that relay signals between the hippocampus and the diencephalon, forebrain, striatum, and prefrontal cortex [40]. Together, the hippocampus and fornix are critical brain regions for making and using memories. In humans, the extent of leukoaraiosis predicts the magnitude of cognitive impairment [41], [42]. Hence, inflammation, disrupted hippocampal neuron homeostasis, and leukoaraiosis are plausible mechanisms for the cognitive impairment we observed in HHcy mice. The idea that BBB leak is a key feature of the etiology of this disease process is supported by our results. Similar changes may mediate the association between HHcy and cognitive decline in humans.
